# Leukocytes telomere length as a biomarker of adverse drug reactions induced by Osimertinib in advanced non-small cell lung cancer

**DOI:** 10.1038/s41598-024-77935-0

**Published:** 2024-11-03

**Authors:** Narumol Trachu, Thanyanan Reungwetwattana, Jennis Meanwatthana, Chonlaphat Sukasem, Teerapat Majam, Wacharapol Saengsiwaritt, Jiraphun Jittikoon, Wanvisa Udomsinprasert

**Affiliations:** 1 Research Center, Faculty of Medicine Ramathibodi Hospital, Mahidol University, Bangkok, Thailand; 2 Division of Medical Oncology, Department of Medicine, Faculty of Medicine Ramathibodi Hospital, Mahidol University, Bangkok, Thailand; 3 Department of Pharmacy, Faculty of Pharmacy, Mahidol University, Bangkok, Thailand; 4 Division of Pharmacogenomics and Personalized Medicine, Department of Pathology, Faculty of Medicine Ramathibodi Hospital, Mahidol University, Bangkok, Thailand; 5 School of Pharmacy, Walailak University, Nakhon Si Thammarat, Thailand; 6 Department of Biochemistry, Faculty of Pharmacy, Mahidol University, 447 Sri-Ayudthaya Road, Rajathevi, Bangkok, 10400 Thailand

**Keywords:** Telomere length, Non-small cell lung cancer, Osimertinib, Osimertinib-induced adverse drug reactions, Biomarker, Biomarkers, Diseases, Oncology

## Abstract

This study aimed to measure relative telomere length (RTL) in blood leukocytes of advanced-stage NSCLC patients either with or without Osimertinib-induced ADRs and determine whether RTL could serve as a biomarker of Osimertinib-induced ADRs. Blood leukocytes RTL were measured in 63 advanced-stage NSCLC patients and 62 age-matched healthy controls using real-time polymerase chain reaction. In patients with advanced-stage NSCLC, RTL was significantly shorter than that in healthy controls (*P* < 0.001). Compared to patients without ADRs and those with mild/moderate ADRs, patients with severe ADRs exhibited significantly decreased RTL (*P* < 0.001, *P* < 0.001, respectively). ROC curve analysis uncovered a diagnostic value of RTL as a biomarker of Osimertinib-induced ADRs (AUC = 1.000, *P* < 0.001). Kaplan-Meier analysis revealed a significant association between shorter RTL and increased cumulative incidence of Osimertinib-induced ADRs in patients with advanced-stage NSCLC (*P* < 0.001). Shorter RTL in blood leukocytes would reflect the occurrence of Osimertinib-induced ADRs and might emerge as a promising biomarker for identifying advanced-stage NSCLC patients who are at risk of experiencing Osimertinib-induced ADRs, particularly those with severe ADRs.

**Supplementary Information:**

The online version contains supplementary material available at 10.1038/s41598-024-77935-0.

## Introduction

Targeted therapy is currently considered the established standard-of-care for patients diagnosed with advanced-stage non-small cell lung cancer (NSCLC) who have epidermal growth factor receptor (EGFR) driver mutations, which are the most commonly observed gene mutations in this type of cancer^1,2^. However, the use of EGFR tyrosine-kinase inhibitors (EGFR-TKIs) is often accompanied by the development of drug resistance, resulting in disease progression for all patients over time. The T790M mutation in EGFR exon 20 is the most frequently observed mechanism of resistance to first- and second-generation EGFR-TKI treatment. It has been found in approximately 50–60% of NSCLC patients who were treated with a first- or second-generation EGFR-TKI^3^. Osimertinib, a third-generation EGFR-TKI, has been approved for the first-line treatment of advanced NSCLC and was the initial drug to successfully address the T790M resistance mutation^4^. A Phase III clinical trial has uncovered that the use of a second-line treatment, following initial treatment with a first-generation EGFR-TKI, resulted in a significantly longer progression-free survival (PFS) compared to platinum-pemetrexed chemotherapy^5^. With the increasing clinical use of Osimertinib, the attention towards adverse drug reactions (ADRs) has grown in recent years. Although Osimertinib has been shown to be effective, there have been reports of a relatively high occurrence of ADRs in NSCLC patients receiving Osimertinib^6,7^. These ADRs not only contribute to significant morbidity and mortality rates, but also impose a financial burden on the healthcare system^8^, which could seriously impact the quality of life for affected patients. It is crucial to acknowledge that the identification of rare and serious ADRs may pave the way for accurately and specifically predicting the therapeutic efficacy and safety of antitumor therapy. However, there is, to date, a lack of reliable, specific biomarkers for predicting and monitoring ADRs caused by Osimertinib in patients with NSCLC.

Given that telomeres act as a biological clock determining the lifespan of cells and organisms^9^, telomere length is currently attracting growing attention as a possible biomarker of various pathological conditions^10–20^. Telomeres are repetitive hexanucleotides (TTAGGG)n located at the end of chromosomes^9^. Their main function is to protect chromosome ends from damage and instability^21^. During each cell cycle, telomeres generally undergo shortening, serving as an indicator of the cell’s proliferative history^22^. When telomeres become critically short, cells either enter a state of senescence cell cycle arrest or undergo apoptosis. It has been well-recognized that telomere attrition is primarily influenced by age and genetic factors, but it can also be affected by host-related genetic characteristics, such as gender, as well as lifestyle factors including smoking, physical activity, and stress^23^. From that viewpoint, telomere shortening has been previously linked to various diseases such as lung, liver, hematologic, and cardiovascular diseases, as well as multiple types of cancer^10–24^. In addition to this, alterations in telomere length have been reportedly associated with various types of ADRs^25,26^.

Despite that, to our knowledge, the prognostic value of telomere length in ADRs caused by Osimertinib remains relatively nascent and poorly understood. Accordingly, the objective of this study was to measure telomere length in blood leukocytes of NSCLC patients with and without ADRs and determine whether blood leukocytes telomere length could be utilized as a possible biomarker for identifying the occurrence of severe ADRs in patients with advanced-stage NSCLC.

## Methods

The study protocol received approval from the ethical committee of the Faculty of Dentistry/Faculty of Pharmacy, Mahidol University (MU-MOU COA 2023/029.2504) and was conducted in accordance with the ethical standards specified in the Declaration of Helsinki. All study participants were required to provide written informed consent prior to their involvement in the study.

### Participants and study design

This retrospective and prospective cohort study consisted of 63 patients with advanced-stage NSCLC and 62 age- and gender-matched healthy controls. A cohort of 63 patients with advanced-stage EGFR mutation-positive NSCLC was recruited from the Division of Cancer, Department of Medicine, Faculty of Medicine, Ramathibodi Hospital, Mahidol University. These patients were diagnosed with NSCLC based on histopathological and cytological examinations of primary and metastasis lesions. All patients were administered Osimertinib as a second-line treatment following the failure of first-line chemotherapy or first/second generation EGFR-TKIs as the initial treatment. Healthy volunteers were selected for this study through purposive sampling. These individuals participated in an annual health check-up at Faculty of Medicine Ramathibodi Hospital and met the criteria of having no clinical indications or symptoms of NSCLC, autoimmune disorders, or liver disorders. This information was documented in their medical records and confirmed through consultations.

Inclusion criteria for this study were as follows: a confirmed diagnosis of NSCLC based on histological and cytological evidence, clinical stage IIIB-IV cancer as per the 8th edition of the tumor, node, and metastasis (TNM) classification, presence of activating EGFR mutation, previous treatment with Osimertinib monotherapy, a total white blood cell count of at least 3.5 × 10^9^ /L, an absolute neutrophil count of at least 1.5 × 10^9^ /L, a hemoglobin level of at least 9 g/dL, a platelet count of at least 100 × 10^9^ /L, as well as AST and ALT not exceeding 2.5 times the upper normal limit (UNL), and a total bilirubin level not exceeding 2 mg/dL. All advanced-stage NSCLC patients recruited in our study had TNM stage 4. Patients who were concurrently using other anticancer agents, undergoing thoracic radiotherapy, undergoing postoperative adjuvant chemotherapy, or undergoing second-line treatment were excluded from the study.

The calculation of the sample size was derived from a prospective longitudinal observational cohort study consisting of 53 patients diagnosed with advanced NSCLC who were undergoing Osimertinib therapy^27^. The calculation employed the equation derived from the study conducted by Ngamjarus et al.^28^., as detailed in Supplementary material S1. Based on the calculation for sample size, it is recommended to have a total of 58 participants for the study. In our study, a total of 63 patients with advanced-stage NSCLC were enrolled.

Peripheral blood samples were collected from healthy controls and advanced-stage NSCLC patients who had received treatment with the standard Osimertinib regimen within one day of starting treatment.

### Osimertinib treatment

Following enrollment, all patients received the standard Osimertinib regimen orally, either on an empty stomach or after a low-fat meal. The initial dose administered was 80 mg QDAC, with a reduced dose of 40 mg QDAC being prescribed in the event of level 3 ADRs or long-lasting level 2 ADRs. All patients were given a daily dose of 80 mg of Osimertinib. As of the data cutoff, the average duration of follow-up was 18 months, ranging from 10 to 30 months. It was recommended to permanently discontinue Osimertinib in patients who experienced disease progression or were unable to tolerate the reduced dose of 40 mg QDAC. It was recommended to adhere to a consistent daily schedule when taking the medication. In the event that a patient inadvertently missed a dose, it was permissible to administer the missed dose within a 12-hour timeframe. If patients inadvertently failed to take their medication for a duration exceeding 12 h, it was permissible for them to proceed directly to the subsequent scheduled dose without attempting to compensate for the missed administration. All patients included in this study adhered to the prescribed guidelines and recommended dosage of Osimertinib monotherapy, without any concurrent administration of other medications.

### Assessment of adverse drug reactions (ADRs)

ADRs were systematically evaluated by the physicians of the patients, employing the National Cancer Institute Common Terminology Criteria for Adverse Events (CTCAE) version 5.0, an established and universally recognized standard for the classification and grading of adverse events associated with cancer therapy^29^. It is worth mentioning that ADRs rated as Grade 1 or 2 on the CTCAE scale were considered to be mild or moderate ADRs, whereas those rated as Grade 3 were classified as severe ADRs. The causality assessment regarding the observed ADRs associated with Osimertinib was conducted using the Naranjo algorithm^30^. Information was documented as ADRs if there was categorized as possible, probable, or definite according to the probability scale outlined by the algorithm.

### Quantitative real-time polymerase chain reaction (PCR)

Measurement of relative telomere length (RTL) was conducted using quantitative real-time PCR, following the methodology described in a previous study^31^. Genomic DNA was extracted from peripheral blood leukocytes using the QIAamp DNA Blood Mini Kit (Qiagen, CA, USA), according to the manufacturer’s protocol. PCR was conducted using the Applied Biosystems ViiA 7 eal-Time PCR System (Applied Biosystems, Massachusetts, United States) and Fast SYBE Green Master Mix (Applied Biosystems, Massachusetts, United States). The primers used for amplifying the telomere repeat copy number and the single-copy gene copy number were as follows: telomere forward primer 5′-CGGTTTGTTTGGGTTTGGGTTTGGGTTTGGGTTTGGGTT-3′, telomere reverse primer 5′-GGCTTGCCTTACCCTTACCCTTACCCTTACCCTTACCCT-3′, single-copy gene forward primer 5′-CAGCAAGTGGGAAGGTGTAATCC-3′, and single-copy gene reverse primer 5′-CCCATTCTATCATCAACGGGTACAA-3′. RTL was determined by calculating the ratio of telomere repeat copy number (T) to single-copy gene copy number (S) in each sample. Telomere repeat and single-copy gene quantities were normalized to a reference DNA sample in each sample.

### Statistical analysis

The comparison of normally distributed continuous variables was conducted using Student’s t-test for two groups and ANOVA for more than two groups, followed by a Tukey post hoc test. On the other hand, the comparison of abnormally distributed continuous variables was performed using the Mann-Whitney U test for two groups and the Kruskal-Wallis H test for more than two groups. The chi-square test was employed to assess the presence of statistically significant differences between groups based on categorical variables. Spearman’s Rho correlation was executed to determine the possible correlation between RTL and the severity of Osimertinib-induced ADRs. Receiver Operating Characteristic (ROC) curve was generated to assess the diagnostic utility of blood leukocytes RTL as a potential biomarker for severe ADRs in patients with NSCLC. This analysis provided values for the Area Under the ROC Curve (AUC), sensitivity, and specificity. The data are presented as mean ± standard deviation (SD). To conduct a more comprehensive evaluation of the potential use of blood leukocytes RTL as a prognostic marker for the occurrence of ADRs induced by Osimertinib, Kaplan-Meier curves were generated for all patients categorized as having either shorter or longer RTL, based on the median distribution of RTL in healthy controls. Continuous data are represented as median ± interquartile range (Q1, Q3), while categorical data are represented as both the number and the corresponding percentage. A *P*-value below 0.05 was deemed statistically significant for all analyses. All statistical analyses were conducted using SPSS Statistics version 26.0 (SPSS Inc., Chicago, IL, US) and GraphPad Prism version 9.0 (GraphPad Software, Inc., CA, US).

## Results

### Demographic and clinical characteristics of study participants

A total of 63 patients with advanced-stage NSCLC and 62 healthy controls were included in this study. There were no significant differences observed in median age, gender ratio, and median body mass index (BMI) between patients with advanced-stage NSCLC and healthy controls [median age: 68.00 (60.00, 73.00) vs. 65.00 (58.75, 72.00), *P* = 0.282; gender ratio: 43 (68.25%)/20 (31.75%) vs. 41 (66.13%) / 21 (33.87%), *P* = 0.850; median BMI: 21.73 (19.60, 23.83) vs. 22.37 (20.43, 24.47), *P* = 0.139; respectively]. Based on severity of ADRs, patients with advanced-stage NSCLC were divided into three groups: the patients without ADRs, those with mild/moderate ADRs, and those with severe ADRs. Demographic and clinical characteristics of advanced-stage NSCLC patients with different types of ADRs are summarized in Table 1. No statistically significant differences in median age, gender ratio, median BMI, smoking status, types of EGFR mutations (exon 19 deletion, exon 20 insertion, and G719X), T790M mutation, eastern cooperative oncology group performance status (ECOG PS), and cerebral metastasis status among the patients with different types of ADRs were observed. However, it is worth noting that there was a statistically significant disparity in the frequency of exon 21 L858R point mutation in *EGFR* observed among the patients with different types of ADRs (*P* = 0.014).

**Table 1:** Baseline and clinical characteristics of patients with advanced-stage NSCLC.

Variables	Patients with advanced-stage NSCLC			*P*-values
	No ADRs	Mild/moderate ADRs	Severe ADRs	
Number	6 (9.52%)	27 (42.86%)	30 (47.62)	N/A
Age (years)	69.50 (59.75, 74.50)	68.00 (60.00, 72.00)	68.50 (60.00, 75.00)	0.899
Gender [no. (%)]				
Female	2 (33.33%)	20 (74.07%)	21 (70.00%)	0.147
Male	4 (66.67%)	7 (25.93%)	9 (30.00%)	
BMI (kg/m^2^)	22.04 (17.73, 25.12)	20.81 (19.27, 23.78)	21.76 (20.11, 24.23)	
Smoking status [no. (%)]				
Never	3 (50.00%)	24 (88.89%)	24 (80.00%)	0.89
Ever	3 (50.00%)	3 (11.11%)	6 (20.00%)	
EGFR mutation [no. (%)]				
Exon 19 deletion				
Positive	6 (100.00%)	25 (92.59%)	30 (100.00%)	0.363
Negative	0 (0.00%)	2 (7.41%)	0 (0.00%)	
Exon 20 insertion				
Positive	5 (83.33%)	17 (62.96%)	22 (73.33%)	0.659
Negative	1 (16.67%)	10 (37.04%)	8 (26.67%)	
Exon 21 L858R				
Positive	0 (0.00%)	4 (14.81%)	13 (43.33%)	**0.014**
Negative	6 (100.00%)	23 (85.19%)	17 (56.67%)	
G719X				
Positive	1 (16.67%)	11 (40.74%)	9 (30.00%)	0.424
Negative	5 (83.33%)	16 (59.26%)	21 (70.00%)	
T790M mutation [no. (%)]				
Positive	6 (100.00%)	27 (100.00%)	29 (96.67%)	1.000
Negative	0 (0.00%)	0 (0.00%)	1 (3.33%)	
ECOG PS [no. (%)]				
0	2 (33.33%)	9 (33.33%)	11 (36.67%)	0.782
1	2 (33.33%)	14 (51.85%)	14 (46.67%)	
2	2 (33.33%)	3 (11.11%)	5 (16.67%)	
3 or 4	0 (0.00%)	1 (3.70%)	0 (0.00%)	
Cerebral metastasis [no. (%)]				
No	6 (100.00%)	24 (88.89%)	23 (76.67%)	0.228
Yes	0 (0.00%)	3 (11.11%)	7 (23.33%)	

*ADRs* adverse drug reactions, *BMI* body mass index, *ECOG PS* Eastern cooperative oncology group performance status, *N/A* not available, *NSCLC* non-small cell lung cancer.

*P*-value marked with bold indicates a statistically significant difference among advanced-stage NSCLC patients with different types of ADRs induced by Osimertinib.

Continuous data are represented as median ± interquartile range (Q1, Q3), while categorical data are represented as both the number and the corresponding percentage.

### Overall efficacy of Osimertinib treatment

**Fig. 1:**
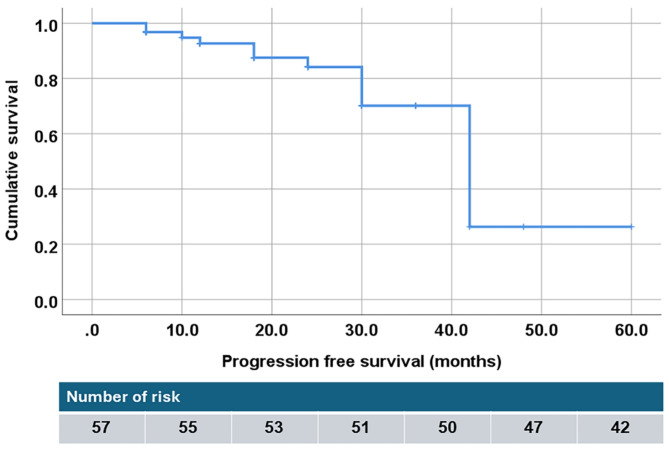
Kaplan–Meier curve for a progression free survival of Osimertinib treatment in advanced-stage NSCLC patients.

The median progression free survival was 18 months, with a range of 0–62 months. Out of 57 patients with advanced-stage NSCLC, the majority, 42 (73.68%), continued their treatment with Osimertinib, while 15 (26.32%) stopped taking Osimertinib due to disease progression, as displayed in Fig. 1.

### ADRs induced by Osimertinib

As shown in Table 2, the most common prevalent ADRs were observed in the skin (acne, 47.37%) and digestive system (diarrhea, 31.58%), followed by QTc prolongation (24.56%), eye-related ADRs (21.05%), blood-related ADRs (thrombocytopenia, 19.30%), and mucositis (12.28%). In terms of the severity of ADRs, advanced-stage NSCLC patients were classified into 2 groups: those with mild/moderate ADRs (grade 1, *n* = 17; grade 2, *n* = 10) and those with severe ADRs (grade 3, *n* = 30).

**Table 2:** Osimertinib-related ADRs in various systems of advanced-stage NSCLC patients.

Variables	Severity of ADRs					
	Overall (*n* = 57)		Mild/moderate (*n* = 27)		Severe (*n* = 30)	
	*n*	%	*n*	%	*n*	%
Severity grading						
1	17	29.83	17	62.96	0	0.000
2	10	17.54	10	37.04	0	0.00
3	30	52.63	0	0.00	30	100.00
Skin						
Dry skin	21	36.84	13	48.15	8	26.67
Alopecia	4	7.02	1	3.70	3	10.00
Acne	27	47.37	15	55.56	12	40.00
Urticaria	7	12.28	3	11.11	4	13.33
Bullous	2	3.51	1	3.70	1	3.33
Papule	2	3.51	0	0.00	2	6.67
Paronychia	8	14.04	5	18.52	3	10.00
Digestive						
Nausea	1	1.75	0	0.00	1	3.33
Anorexia	1	1.75	0	0.00	1	3.33
Diarrhea	18	31.58	9	33.33	9	30.00
Liver						
Transaminitis	5	8.77	2	7.41	3	10.00
Blood						
Anemia	5	8.77	0	0.00	5	16.67
Neutropenia	5	8.77	0	0.00	5	16.67
Thrombocytopenia	11	19.30	0	0.00	11	36.67
Dry eye	12	21.05	6	22.22	6	20.00
Mucositis	7	12.28	2	7.41	5	16.67
Myalgia	3	5.26	1	3.70	2	6.67
Myositis	2	3.51	1	3.70	1	3.33
Prolonged QTc intervals	14	24.56	2	7.41	12	40.00

*ADRs* adverse drug reactions, *NSCLC* non-small cell lung cancer.

In the patients with mild/moderate ADRs, the main manifestation of skin ADRs were acne (55.56%), dry skin (48.15%), paronychia (18.52%), urticaria (11.11%), alopecia (3.70%), and bullous (3.70%). Diarrhea was the most common type of ADRs in the digestive system, constituting 33.33% of advanced-stage NSCLC patients with mild/moderate ADRs. The most prevalent ADR related to the eyes was dry eye, accounting for 22.22% of cases. Liver-related ADRs included transaminitis, which was observed in 7.41% of cases. Additionally, the patients with mild/moderate ADRs exhibited clinical symptoms including mucositis (7.41%), QTc prolongation (7.41%), myalgia (3.70%), and myositis (3.70%).

Severe ADRs were observed in 30 advanced-stage NSCLC patients. These reactions specifically affected the skin system, with the following frequencies: acne (40.00%), dry skin (26.67%), urticaria (13.33%), alopecia (10.00%), papule (6.67%), and bullous (3.33%). The most prevalent ADR observed was QTc prolongation, which accounted for 40.00% of reported cases. Among patients with advanced-stage NSCLC experiencing severe ADRs in the digestive system, diarrhea was found to be the most commonly reported symptom, accounting for 30.00% of the observed cases. Nausea and anorexia were found to be the subsequent most common ADRs, each occurring in 3.33% of patients. Thrombocytopenia was the most common type of blood-related ADRs, constituting 36.67% of advanced-stage NSCLC patients with severe ADRs, followed by anemia (16.67%) and neutropenia (16.67%). In addition to this, the patients with severe ADRs clinically manifested mucositis (16.67), transaminitis (10.00%), myalgia (6.67), and myositis (3.33%).

### Blood leukocytes RTL in healthy controls and patients with advanced-stage NSCLC

**Fig. 2:**
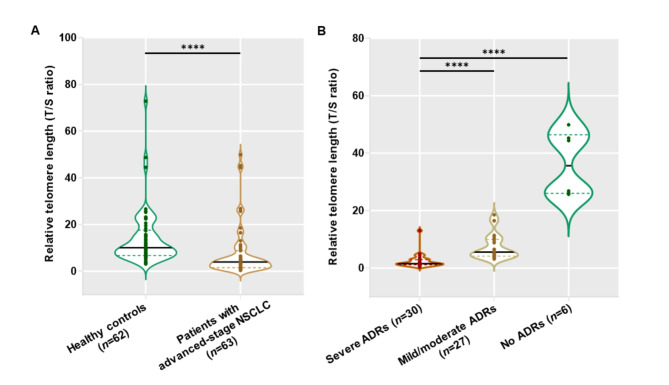
RTL in blood leukocytes of healthy controls and advanced-stage NSCLC patients with and without ADRs induced by Osimertinib. (**A**) Comparison in blood leukocytes RTL between healthy controls and patients with advanced-stage NSCLC. (**B**) Comparison in blood leukocytes RTL among the patients with different types of Osimertinib-induced ADRs. ****P < 0.0001.

Blood leukocytes RTL were initially compared among advanced-stage NSCLC patients with and without ADRs, as well as unaffected control individuals. Blood leukocytes RTL were observed to be significantly shorter in patients with advanced-stage NSCLC than that in healthy controls [3.94 (1.52, 8.79) vs. 10.07 (6.74, 17.60), *P* < 0.0001] (Fig. 2A). In stratified analysis based on severity of ADRs caused by Osimertinib, it was found that patients with advanced-stage NSCLC who experienced severe ADRs exhibited significantly shorter blood leukocytes RTL than those with mild/moderate ADRs [1.52 (1.12, 2.91) vs. 5.55 (4.14, 9.96), *P* < 0.0001] (Fig. 2B). Consistent with this, the patients with severe ADRs had a significant reduction in blood leukocytes RTL compared to those without ADRs [1.52 (1.12, 2.91) vs. 35.61 (26.01, 46.43), *P* < 0.0001] (Fig. 2B). In addition, there was a statistically significant disparity observed in blood leukocytes RLT between the patients experiencing mild/moderate ADRs and those without ADRs [5.55 (4.14, 9.96) vs. 35.61 (26.01, 46.43), *P* < 0.0001] (Fig. 2B).

To further determine the possible correlation between blood leukocytes RTL and severity of Osimertinib-induced ADRs, Spearman’s Rho correlation analysis was executed. The analysis uncovered that blood leukocytes RTL were negatively correlated with the severity of Osimertinib-induced ADRs in advanced-stage NSCLC patients (*r* = − 0.82, *P* < 0.0001).

### Diagnostic value of blood leukocytes RTL as a biomarker of Osimertinib-induced ADRs

**Fig. 3:**
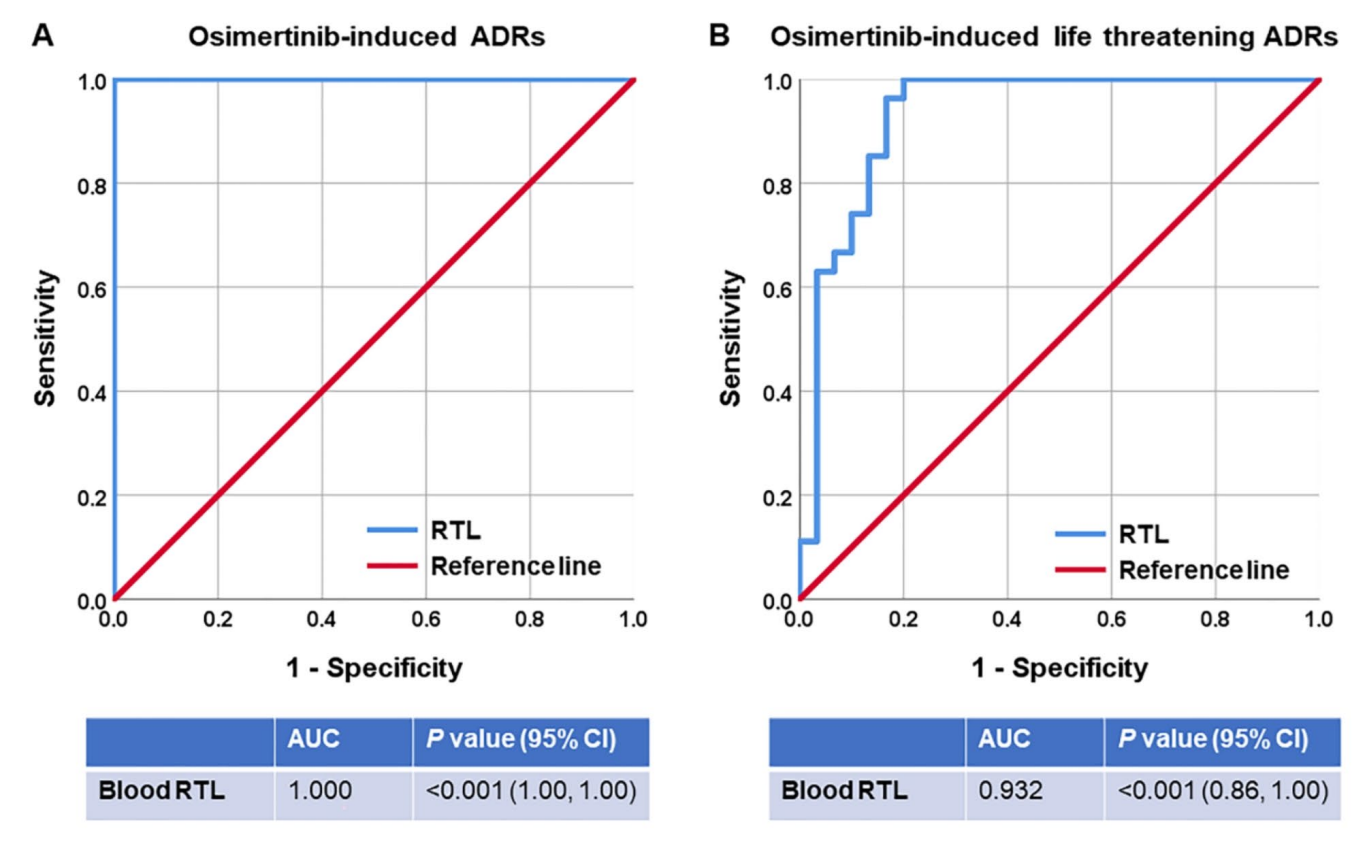
ROC analysis of blood leukocytes RTL as a diagnostic biomarker of Osimertinib-induced ADRs in patients with advanced-stage NSCLC. (**A**) Blood leukocytes RTL as a biomarker for discriminating patients with advanced-stage NSCLC who experienced Osimertinib-induced ADRs from those without ADRs. (**B**) Blood leukocytes RTL as a biomarker for distinguishing patients with advanced-stage NSCLC who experienced Osimertinib-induced severe ADRs from those with mild/moderate ADRs.

To assess the potential utility of blood leukocytes RTL as a biomarker for identifying development of Osimertinib-induced severe ADRs in patients with advanced-stage NSCLC, the AUC was calculated. ROC curve analysis uncovered that the optimal cutoff value of blood leukocytes RTL as a biomarker for patients with advanced-stage NSCLC who experienced ADRs induced by Osimertinib from those patients without ADRs was defined at 22.090, which yielded a sensitivity of 100.0%, a specificity of 100.0%, and an AUC of 1.00 (95% CI 1.00–1.00; *P* < 0.0001) (Fig. 3A). For discriminating patients with advanced-stage NSCLC who experienced severe ADRs induced by Osimertinib from those patients with mild/moderate ADRs, blood leukocytes RTL of 3.055 provided a sensitivity of 100.0%, a specificity of 80.0%, and an AUC of 0.932 (95% CI 0.86–1.00; *P* < 0.0001) (Fig. 3B).

### Association between blood leukocytes RTL and a cumulative incidence of Osimertinib-induced ADRs

**Fig. 4:**
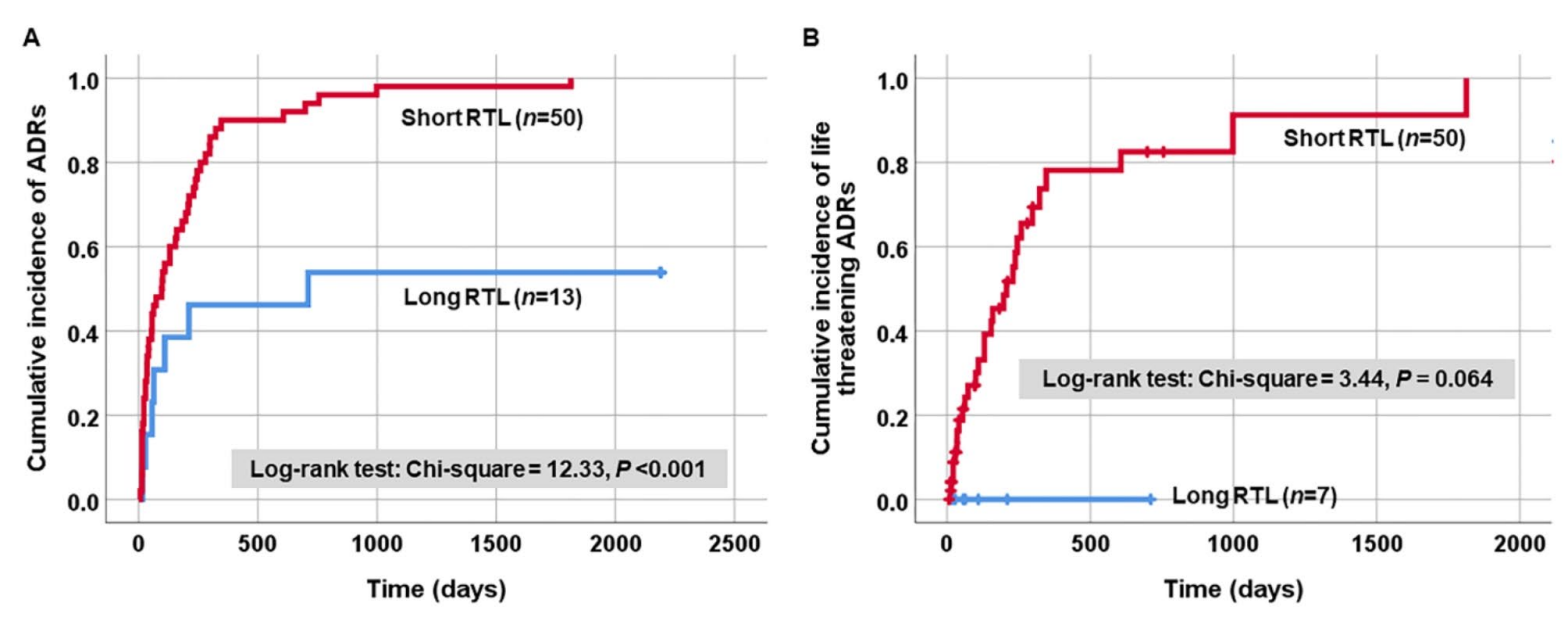
Kaplan–Meier curve of blood leukocytes RTL as prognostic biomarker of Osimertinib-induced ADRs in patients with advanced-stage NSCLC. (**A**) A significant association between shorter RLT in blood leukocytes and increased cumulative rate of Osimertinib-induced ADRs. (**B**) No association between shorter RLT in blood leukocytes and increased cumulative rate of Osimertinib-induced severe ADRs.

Given shorter RTL as an independent determinant of Osimertinib-induced ADRs, the effect of shorter RTL on a cumulative incidence of ADRs occurrence in patients with advanced-stage NSCLC was additionally determined. The Kaplan-Meier analysis revealed a statistically significant increase in cumulative incidence of ADRs induced by Osimertinib in patients with advanced-stage NSCLC who had shorter RTL compared to those with longer RTL (log-rank: Chi-square = 12.33, *P* < 0.001) (Fig. 4A). Furthermore, NSCLC patients with shorter RTL exhibited a higher cumulative incidence of severe ADRs induced by Osimertinib compared to those with longer RTL. However, it is important to note that this difference did not reach statistical significance (log-rank: Chi-square = 3.44, *P* = 0.064) (Fig. 4B).

## Discussion

It has been well-recognized that ADRs are a major public health concern, thereby emphasizing the importance of early detection for both drug development and patient safety. In tumor treatment, biomarkers have been extensively utilized to enable precise and reliable prediction of therapeutic effectiveness and safety throughout antitumor therapy. This allows oncologists to promptly adjust the treatment plan in order to provide the most optimal therapy for their patients^32^. This study evaluated the relationship between blood leukocytes RTL and ADRs caused by Osimertinib in patients with advanced-stage NSCLC and observed that shorter RTL was associated with increased likelihood of ADRs. Our subsequent analysis confirmed the aforementioned finding by demonstrating that patients with advanced-stage NSCLC who had shorter RTL experienced a significantly higher cumulative incidence of ADRs after receiving Osimertinib. From our above-mentioned findings, it has been hypothesized that telomere shortening might be implicated in the developmental and progressive ADRs induced by Osimertinib in patients with advanced-stage NSCLC, predominantly those patients with severe ADRs.

Although there is no clear link between changes in blood leukocytes RTL and ADRs caused by antitumor therapy, numerous published studies have provided evidence of alterations in blood leukocytes RTL in ADR induced by anti-tuberculosis drug including DILI^25,26^. In the realm of multifactorial diseases, several clinical studies have uncovered connections between shorter RTL and reduced lung function, as well as an increased risk of chronic obstructive pulmonary disease^33,34^. Apart from determination on RTL in chronic inflammatory lung disease, our previous research has demonstrated a consistent reduction in RTL in patients with chronic liver disease like biliary atresia (BA), particularly those with advanced-stage^35^. In terms of lung cancer, there is growing evidence revealing a strong association between telomere shortening and an increased risk of cancer development^36,37^. The foregoing findings are all comparable with our own research and offer additional evidence to support the notion that RTL in blood leukocytes could serve as a reliable biomarker for monitoring the occurrence of Osimertinib-induced ADRs in patients with advanced-stage NSCLC. In support of this speculation, our additional findings obtained from ROC curve analysis revealed that blood leukocytes RTL could be used as a sensitive, specific biomarker for identifying Osimertinib-induced ADRs. Concurrently, this study also examined the effect of shorter RTL on cumulative incidence of Osimertinib-induced ADRs. The findings revealed a strong association between shorter RTL and an increased likelihood of Osimertinib-induced ADRs in patients with advanced-stage NSCLC. Our findings collectively provide insight into the potential utilization of blood leukocytes RTL as a diagnostic biomarker for the occurrence of Osimertinib-induced ADRs in patients with advanced-stage NSCLC.

Although the precise mechanisms underlying the relationship between shorter RTL and an increased incidence of Osimertinib-induced ADRs remain undefined, one possible explanation for shorter RTL in blood leukocytes of patients with advanced-stage NSCLC, especially those with severe ADRs due to Osimertinib, might be attributed to dysfunction of immune cells, leading to oxidative DNA damage by reactive oxygen species (ROS) and ultimately to shortened RTL in blood leukocytes. This assumption has been substantiated by previous studies showing that drug resistance could also arise from an enhanced translocation of the telomerase hTERT (telomerase reverse transcriptase) subunit to mitochondria, or the translocation of another telomerase-associated factor, telomerase-associated protein 1 (TEP1), to vaults. It has been reported that mitochondrial enrichment with hTERT potentially influences ROS production^38,39^. To substantiate this, there exists a substantial body of documentation denoting a direct link between high levels of ROS and shortened telomeres in blood leukocytes^40–42^. Based on these perspectives, it is conceivable that patients with advanced-stage NSCLC who possessed shortened telomeres might be more susceptible to accumulative production of ROS, and thus at an increased likelihood of experiencing ADRs induced by Osimertinib, especially those of severe ADRs. Considering a notable reduction in blood leukocytes RTL in patients with advanced-stage NSCLC compared to healthy controls, it is worth contemplating the potential explanation that shorter telomeres observed in blood leukocytes of advanced-stage NSCLC patients could be due to the impairment of immune cell function, which possibly accelerates the process of immune senescence^43–45^. Based on the available evidence, it is reasonable to infer that NSCLC patients with shorter RLT might be more prone to premature immune senescence, potentially increasing their vulnerability to lung cancer. However, future studies are necessary to elucidate the mechanism through which telomere shortening contributes to the increased susceptibility of Osimertinib-induced ADRs and advanced-stage NSCLC.

Notwithstanding the findings presented in this study, it is important to acknowledge certain inherent limitations that should be duly considered. One limitation is the unavailability of RTL measurement in tissue-specific cancerous cells from advanced-stage NSCLC patients with and without Osimertinib-induced ADRs. Nevertheless, there exist published data that provide evidence of substantial changes in blood leukocytes RTL content among patients with lung cancer^34,35^. These findings lend support to the notion that alterations in blood leukocytes RTL might serve as an indicator of the occurrence of NSCLS and Osimertinib-induced ADRs. One additional concern pertains to the insufficient availability of data regarding the co-occurrence of other medical conditions with ADRs induced by Osimertinib, as well as the immune-metabolic profile in blood leukocytes. It becomes challenging to accurately interpret our findings regarding the independent association between shorter RTL and an increased risk of Osimertinib-induced ADRs in patients with advanced-stage NSCLC. Furthermore, given that our primary study was to measure RTL in blood leukocytes of advanced-stage NSCLC patients who exclusively received Osimertinib, it becomes challenging to generalize our findings to other TKIs. Besides this, the present study had limitations in terms of comparing blood leukocytes RTL between advanced-stage NSCLC patients with and without Osimertinib therapy. Furthermore, it should be noted that this study is of a cross-sectional design and has a limited sample size. For this reason, it is not feasible to draw definitive conclusions regarding the causal relationship between blood leukocytes RTL and Osimertinib-induced ADRs in patients with advanced-stage NSCLC. Besides this, considering the impracticality of incorporating new samples or external data for the validation of our findings, a limited sample size might introduce sampling error, which could ultimately result in inaccuracies or distortions in the research outcomes. It is crucial to emphasize that the results of this study need to be confirmed with a larger sample size. As individuals age, telomeres naturally undergo a process of shortening. In this study, both the clinical patient group and the healthy control group comprised individuals who were approximately 69 years old, at which point telomere length would typically be relatively short. Consequently, the diagnostic indicator value of RTL for ADRs caused by Osimertinib in NSCLC might not accurately represent the situation across various age groups. It is important to consider whether studying populations from different age groups should be done to ensure the broad applicability of these findings. It is important to note that enhancing the reliability of our conclusions could be achieved by incorporating data from other databases such as the Cancer Genome Atlas (TCGA) or Gene Expression Omnibus (GEO). Without data on the baseline RTL in blood leukocytes for each patient, it becomes challenging to make a direct comparison of the RTL in blood leukocytes before and after receiving Osimertinib treatment. Considering the intricate and diverse nature of ADRs, it would be beneficial to evaluate RTL in conjunction with other plasma-based biomarkers and relevant clinical indicators. This approach could lead to the development of a comprehensive predictive biomarker, enhancing the accuracy and practicality of assessing ADRs.

This study is the first to provide evidence that RTL in blood leukocytes of patients with advanced-stage NSCLC was significantly shorter than that of healthy controls. In comparison to those patients without Osimertinib-induced ADRs, patients with advanced-stage NSCLC who experienced Osimertinib-induced ADRs exhibited a significant decrease in RTL, particularly those with severe ADRs. Further analysis of ROC curve uncovered a diagnostic value of blood leukocytes RTL as a possible biomarker for monitoring the occurrence of ADRs caused by Osimertinib. More specifically, shorter RTL was shown to be significantly associated with an increased cumulative incidence of Osimertinib-induced ADRs. Based on our research findings, it appears that blood leukocytes RTL could serve as a potentially valuable biomarker for Osimertinib-induced ADRs in patients with advanced-stage NSCLC. To verify our results presented herein, it is imperative to conduct a prospective cohort study with larger sample sizes.

## Electronic Supplementary Material

Below is the link to the electronic supplementary material.

Supplementary Material 1

## Data Availability

The data that support the findings of this study are available from the corresponding author upon reasonable request. Some data may not be made available because of privacy or ethical restrictions.
