# Supplementary material for: Leukocytes telomere length as a biomarker of adverse drug reactions induced by Osimertinib in advanced non-small cell lung cancer
Source: Sci Rep. 2024 Nov 3;14:26543. doi: 10.1038/s41598-024-77935-0 (PMC11532503; doi:10.1038/s41598-024-77935-0)
Supplement: Supplementary file 1 — Supplementary Material 1 [file 41598_2024_77935_MOESM1_ESM.docx]

**Supplementary material**

**Sample size calculation**

The calculation of the sample size was derived from a prospective longitudinal observational cohort study consisting of 53 patients diagnosed with advanced NSCLC who were undergoing Osimertinib therapy.^16^ The calculation employed the equation derived from the study conducted by Ngamjarus et al.,^17^ as detailed below. Based on the calculation for sample size, it is recommended to have a total of 58 participants for the study. In our study, a total of 63 patients with advanced-stage NSCLC were enrolled.


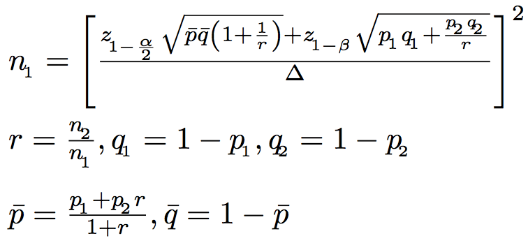


Proportion in group1 (p₁) = 0.6875

Proportion in group2 (p₂) = 1.0

ratio (r) = 22/3 = 7.3

Alpha (α) = 0.05

Beta (β) = 0.20

The sample size was 58 patients.
